# Epigenetic variation for agronomic improvement: an opportunity for vegetatively propagated crops

**DOI:** 10.1002/ajb2.1357

**Published:** 2019-09-10

**Authors:** Mathieu Latutrie, Delphine Gourcilleau, Benoit Pujol

**Affiliations:** ^1^ PSL Université Paris: EPHE‐UPVD‐CNRS USR 3278 CRIOBE Université de Perpignan 52 Avenue Paul Alduy 66860 Perpignan Cedex France; ^2^ Laboratoire Évolution & Diversité Biologique (EDB, UMR 5174) Université Fédérale de Toulouse Midi‐Pyrénées, CNRS, IRD, UPS. 118 route de Narbonne, Bat 4R1 31062 Toulouse cedex 9 France

**Keywords:** crop breeding, epibreeding, epigenetic variation, vegetatively propagated crops

## WHY IS EPIGENETICS OF INTEREST TO PLANT BREEDERS?

Crop breeding has been historically associated with genetic variation because genetic variants harboring traits with agronomic value can be artificially generated (e.g., hybrid crosses) and selected at the population (e.g., mass selection) or individual level (e.g., single seed descent) through classical crop breeding methods. Crop genetic improvement, however, has become increasingly difficult, and crop genetic diversity has been declining at an alarming rate, threatening world food security and sustainable development (Esquinas‐Alcázar, 2005). The stakes are high to find solutions. Cross breeding with closely related wild species, genomic selection informed by molecular markers and creation of genetically modified organisms are examples of ongoing research developments for crop improvement. Another potential research approach that is currently arising is epigenetics (Kenchanmane Raju and Niederhuth, 2018). Epigenetic variants in crops might represent an additional and timely resource for crop breeding. In this essay, we do not aim to comprehensively review the potential interest of epigenetics in agriculture but to outline the nature of some possible drawbacks, such as the instability of epigenetic marks, and opportunities, for example, clonal multiplication, associated with this potential.

## EPIGENETIC VARIANTS OF INTEREST FOR CROP BREEDING: WHERE ARE WE NOW?

Changes in gene expression that are independent of changes in the DNA sequence fall into the category of epigenetic variation (Kapazoglou et al., 2018). Mechanisms of interest include, for example, chromatin rearrangements (heterochromatin vs. euchromatin) that can be associated with DNA methylation changes and histone modifications (Kenchanmane Raju and Niederhuth, 2018). Natural epigenetic variants exist, as for example in toadflax (*Linaria vulgaris*), where the peloric epimutant bears actinomorphic flowers rather than normally zygomorphic flowers (Cubas et al., 1999). It is also possible to generate divergent DNA methylomes that share a similar genomic background and harbor different traits by mutagenesis. However, such stable trait epigenetic variants have only been created to date for the model species *Arabidopsis thaliana* (Cortijo et al., 2014).

Epigenetic variants with potential agronomic interest that have been identified in several crop species include, for example, dwarf phenotypes in rice (Chen and Zhou, 2013), anthocyanin production in apple (Telias et al., 2011), an increased seed protein content and decreased oil content in oilseed rape (Long et al., 2011), sex determination in melon (Martin et al., 2009), and fruit ripening in tomato (Liu et al., 2015). Epigenetic variants of agronomic interest can also be obtained by using chemical treatments such as the exposure to the methylation inhibitor 5‐azacytidine, e.g., early flowering in strawberry (Xu et al., 2016). Locus‐specific epigenome editing techniques such as transcription activator‐like effectors nucleases (TALENs), zinc fingers nucleases (ZFNs) or CRISPR‐Cas9 (Hung et al., 2018) might also be used in the near future. Caution must nevertheless be taken because targeted genes with strong effects might be involved in multiple pathways. Their modification might therefore have complex unexpected pleiotropic effects. Natural environmental stressors can also be used as a source of epigenetic variation for physiological traits in crops, e.g., drought and salt tolerance in rice (Garg et al., 2015). Collectively, all these methods illustrate significant progress in the identification and generation of epigenetic variants for traits of agronomic interest. The use of epigenetic variation for crop improvement, termed plant epibreeding, therefore has potential (Kapazoglou et al., 2018).

## CLONALLY PROPAGATING CROP EPIGENETIC VARIANTS FOR STABLE ADVANTAGES

One major issue must be addressed before epibreeding can be practically implemented. The stable inheritance of trait epigenetic variants is a necessary condition. The transmission of epigenetic marks to meiotic descendants through sexual reproduction is unstable (Danchin et al., 2019) because some DNA methylation changes and histone modifications are often reset during meiosis. In contrast, clonally propagated plants do not undergo meiosis and gametogenesis. Furthermore, the transmission of epigenetic marks through mitosis appears to be stable. As a result, the transmission of epigenetic variants to the next generation in clonally multiplied plants (e.g., cuttings, in vitro propagation) is stable. There is evidence of this stability lasting up to five rounds of clonal propagation, such as genomewide DNA methylation modifications associated with biomass changes induced by maternal stress (drought, soil contamination, and shading) in the clonal plant *Trifolium repens* L. (Rendina González et al., 2018) and global demethylation associated with early flowering in the clonally propagated plant *Fragaria vesca* (Xu et al., 2016).

Clonal propagation has proven efficient for the large‐scale reproduction of genotypes with advantageous combinations of genes that would have been lost if they had undergone the recombination stage of sexual reproduction (McKey et al., 2010). We can draw a parallel between this clonal propagation of genotypes and the clonal propagation of epigenetic variants that provides an opportunity to multiply stable epigenomes harboring traits of interest over many generations. In polyploid species, duplicated epigenomes in autopolyploids and the combination of different epigenomes in allopolyploids are expected to be associated with a broader range of traits as a result of epigenetic redundancy and interactions between epigenomes. Clonal propagation might also allow fixing advantageous traits generated by this type of epigenomic interactions. Meyer et al. (2012) estimated for 203 crops that 39.9% reproduce exclusively by seeds, 9% by clonal propagation, and 51.8% have a mixed reproductive system (both seed and clonal). Thus, over 60% of crops may be amenable to epibreeding because of the stability offered by the clonal multiplication of epigenetic variants. Yet to date, epigenetic studies of vegetatively propagated crops remain surprisingly scarce. Furthermore, the stakes are high for epibreeding in clonally propagated crops because they feed a large proportion of the world (Table 1). They also play an important role in subsistence farming and provide benefits for small farmers in many poor countries.

**Table 1:** Summary data of 2017 crop production in the world.

Crop	Type of reproduction	Area harvested (10^6^ ha)	Yield (t/ha)	Production (10^6^ t)	Ploidy level	Main consumers
Maize	SF / O	197.19	5.75	1134.75	2*x*	USA/China/Brasil
Wheat	SF	218.54	3.53	771.72	6*x*	China/India/Russia
Rice	SF	167.25	4.6	769.66	2*x*	China/India/Indonesia
**Potatoes**	**O / Veg**	**19.3**	**20.11**	**388.19**	**4** ***x***	**China/India**/Russia
Soybean	SF	123.55	2.85	352.64	2*x*	China/USA/Brasil
**Cassava**	**SF / Veg**	**26.34**	**11.08**	**291.99**	**2** ***x***	**Nigeria/China/Indonesia**
**Sweet potatoes**	**Veg**	**9.2**	**12.26**	**112.84**	**2** ***x*** **, 4** ***x*** **, 6** ***x***	**China/Nigeria/Tanzania**
**Yams**	**O / Veg**	**8.56**	**8.53**	**73.02**	**4** ***x*** **, 6** ***x*** **, 8** ***x***	**Nigeria/Ghana/Ivory Coast**
Sorghum	SF / Veg	40.67	1.42	57.6	2*x*	Mexico/Nigeria/India
**Plantains**	**Veg**	**5.52**	**7.11**	**39.24**	**2** ***x*** **, 3** ***x*** **, 4** ***x***	**Uganda/Cameroun/Ghana**

Modified from FAOSTAT (http://www.fao.org/faostat/en/#data/QC). Type of reproduction: O, outcrossing; SF, self‐fertilization; Veg, vegetative propagation (Meyer et al., 2012); in bold: vegetatively propagated corps.

An important aspect to consider is that around 50% of crop species can reproduce both sexually and clonally. Vegetative propagation of otherwise seed‐propagated plants might be an opportunity to overcome at least partially the issue of their between‐generation lack of stability of epigenetic variation. Epigenetic variants with agronomic interest might be generated in plants originating from seeds before being vegetatively propagated by grafting, cuttings, and plant tissue culture (Springer and Schmitz, 2017). More studies testing this hypothesis are needed because clonal popagation might be a great opportunity for the development of epibreeding in a greater number of plants.

## EPIBREEDING

**Figure 1:**
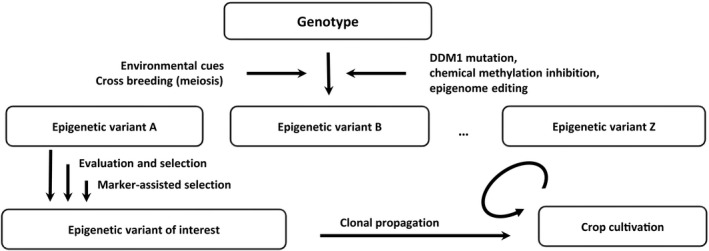
Epibreeding design. After epigenetic variation is generated, variants of interest are chosen and propagated clonally.

Epibreeding does not require selection methods that would differ in nature from conventional crop breeding methods. Conventional approaches based on genetic variation (e.g., cross breeding followed by selection) can be transferred to epibreeding with the obvious differences in terms of epigenetic variant induction, production, and propagation that were discussed earlier. The result of classical crop breeding methods applied to standing, environmentally or artificially induced, epigenetic variation, might be stabilized when necessary by using vegetative propagation (Fig. 1). Selection trials might be assisted by using molecular epigenetic markers, as is already done with genetic markers. Epigenomic marks associated with phenotypic variants of interest have already been identified. For example, differentially methylated regions (DMRs) were used for the early detection of hypomethylated genes associated with the unwanted oil palm fruit “mantled” abnormal phenotype (catastrophic homeotic transformation, parthenocarpy and marked loss of yield) (Ong‐Abdullah et al., 2015).

## ON THE NATURE OF EPIBREEDING

Here we call for more studies on clonally propagated crops such as yam, cassava, sweet potato, taro, and potato (see Table S1 of McKey et al., 2010 for a broader list), for which epigenetic stability would not be a huge concern. These high stake crops are good candidates for epibreeding approaches. Epigenetic variants of interest might even already exist, yet remain undescribed, in some of these plants as a result of their history of clonal selection. New variants could also be generated by using the molecular biology toolkit that already exists. Although still challenging to date, the characterization of epigenetic variation is necessary for the implementation of epibreeding in these crops. Epibreeding appears not so different in nature from genetic breeding in clonally multiplied plants. Increased access to molecular tools for creating and identifying variants of agronomic interest for nonmodel species is a limiting factor. Epigenetic characterization of variants at the scale of populations, which is necessary for epibreeding, is also limiting. These population level approaches lack precision (Gourcilleau et al., 2019). Technological progress is fast, and these problems will soon be solved. There is no doubt that clonally propagated crop epibreeding will gain interest and take off.

## References

[ajb21357-bib-0001] Chen, X. , and D.‐X. Zhou . 2013 Rice epigenomics and epigenetics: challenges and opportunities. Current Opinion in Plant Biology 16: 164–169. 23562565 10.1016/j.pbi.2013.03.004

[ajb21357-bib-0002] Cortijo, S. , R. Wardenaar , M. Colomé‐Tatché , A. Gilly , M. Etcheverry , K. Labadie , E. Caillieux , et al. 2014 Mapping the epigenetic basis of complex traits. Science 343: 1145–1148. 24505129 10.1126/science.1248127

[ajb21357-bib-0003] Cubas, P. , C. Vincent , and E. Coen . 1999 An epigenetic mutation responsible for natural variation in floral symmetry. Nature 401: 157–161. 10490023 10.1038/43657

[ajb21357-bib-0004] Danchin, E. , A. Pocheville , O. Rey , B. Pujol , and S. Blanchet . 2019 Epigenetically facilitated mutational assimilation: epigenetics as a hub within the inclusive evolutionary synthesis. Biological Reviews 94: 259–282.

[ajb21357-bib-0005] Esquinas‐Alcázar, J. 2005 Protecting crop genetic diversity for food security: political, ethical and technical challenges. Nature Reviews Genetics 6: 946–953. 10.1038/nrg1729 16341075

[ajb21357-bib-0006] Garg, R. , V. V. S. Narayana Chevala , R. Shankar , and M. Jain . 2015 Divergent DNA methylation patterns associated with gene expression in rice cultivars with contrasting drought and salinity stress response. Scientific Reports 5: 14922. 26449881 10.1038/srep14922 PMC4598828

[ajb21357-bib-0007] Gourcilleau, D. , M. Mousset , M. Latutrie , S. Marin , A. Delaunay , S. Maury , and B. Pujol . 2019 Assessing global DNA methylation changes associated with plasticity in seven highly inbred lines of snapdragon plants (*Antirrhinum majus*). Genes 10: 256. 10.3390/genes10040256 PMC6523709 30925802

[ajb21357-bib-0008] Hung, Y.‐H. , F. Liu , X.‐Q. Zhang , W. Xiao , and T.‐F. Hsieh . 2018 Sexual and non‐sexual reproduction: inheritance and stability of epigenetic variations and consequences for breeding application *In* Mirouze M., Bucher E., and Gallusci P. [eds.], Advances in Botanical Research, vol. 88, 117–163. Academic Press, London, UK.

[ajb21357-bib-0009] Kapazoglou, A. , I. Ganopoulos , E. Tani , and A. Tsaftaris . 2018 Epigenetics, epigenomics and crop improvement *In* Kuntz M. [ed.], Advances in Botanical Research, vol. 86, 287–324. Academic Press, London, UK.

[ajb21357-bib-0010] Kenchanmane Raju, S. K. , and C. E. Niederhuth . 2018 Epigenetic diversity and application to breeding *In* Mirouze M., Bucher E., and Gallusci P. [eds.], Advances in Botanical Research, vol. 88, 49–86. Academic Press, London, UK.

[ajb21357-bib-0011] Liu, R. , A. How‐Kit , L. Stammitti , E. Teyssier , D. Rolin , A. Mortain‐Bertrand , S. Halle , et al. 2015 A DEMETER‐like DNA demethylase governs tomato fruit ripening. Proceedings of the National Academy of Sciences, USA 112: 10804–10809. 10.1073/pnas.1503362112 PMC4553810 26261318

[ajb21357-bib-0012] Long, Y. , W. Xia , R. Li , J. Wang , M. Shao , J. Feng , G. J. King , and J. Meng . 2011 Epigenetic QTL mapping in *Brassica napus* . Genetics 189: 1093–1102. 21890742 10.1534/genetics.111.131615 PMC3213370

[ajb21357-bib-0013] Martin, A. , C. Troadec , A. Boualem , M. Rajab , R. Fernandez , H. Morin , M. Pitrat , et al. 2009 A transposon‐induced epigenetic change leads to sex determination in melon. Nature 461: 1135–1138. 19847267 10.1038/nature08498

[ajb21357-bib-0014] McKey, D. , M. Elias , B. Pujol , and A. Duputié . 2010 The evolutionary ecology of clonally propagated domesticated plants. New Phytologist 186: 318–332. 20202131 10.1111/j.1469-8137.2010.03210.x

[ajb21357-bib-0015] Meyer, R. S. , A. E. DuVal , and H. R. Jensen . 2012 Patterns and processes in crop domestication: an historical review and quantitative analysis of 203 global food crops. New Phytologist 196: 29–48. 22889076 10.1111/j.1469-8137.2012.04253.x

[ajb21357-bib-0016] Ong‐Abdullah, M. , J. M. Ordway , N. Jiang , S.‐E. Ooi , S.‐Y. Kok , N. Sarpan , N. Azimi , et al. 2015 Loss of *Karma* transposon methylation underlies the mantled somaclonal variant of oil palm. Nature 525: 533–537. 26352475 10.1038/nature15365 PMC4857894

[ajb21357-bib-0017] Rendina González, A. P. , V. Preite , K. J. F. Verhoeven , and V. Latzel . 2018 Transgenerational effects and epigenetic memory in the clonal plant *Trifolium repens* . Frontiers in Plant Science 9: 1667. 30524458 10.3389/fpls.2018.01677 PMC6256281

[ajb21357-bib-0018] Springer, N. M. , and R. J. Schmitz . 2017 Exploiting induced and natural epigenetic variation for crop improvement. Nature Reviews Genetics 18: 563. 10.1038/nrg.2017.45 28669983

[ajb21357-bib-0019] Telias, A. , K. Lin‐Wang , D. E. Stevenson , J. M. Cooney , R. P. Hellens , A. C. Allan , E. E. Hoover , and J. M. Bradeen . 2011 Apple skin patterning is associated with differential expression of MYB10. BMC Plant Biology 11: 93. 21599973 10.1186/1471-2229-11-93 PMC3127826

[ajb21357-bib-0020] Xu, J. , K. K. Tanino , and S. J. Robinson . 2016 Stable epigenetic variants selected from an induced hypomethylated *Fragaria vesca* population. Frontiers in Plant Science 7: 1768. 27965682 10.3389/fpls.2016.01768 PMC5126047

